# The Role of Endoplasmic Reticulum Stress and NLRP3 Inflammasome in Liver Disorders

**DOI:** 10.3390/ijms23073528

**Published:** 2022-03-24

**Authors:** Xueqin Lu, Haitao Huang, Xiaodi Fu, Chaoran Chen, Huiyang Liu, Honggang Wang, Dongdong Wu

**Affiliations:** 1 Institute of Nursing and Health, School of Nursing and Health, Henan University, Jinming Avenue, Kaifeng 475004, China; luxueqin2010@163.com (X.L.); huangtaomiss@163.com (H.H.); kfccr@henu.edu.cn (C.C.); 2 School of Basic Medical Sciences, Henan University, Kaifeng 475004, China; fuxiaodi2020@163.com (X.F.); m15736875597@163.com (H.L.); 3 School of Stomatology, Henan University, Kaifeng 475004, China; 4 Henan International Joint Laboratory of Nuclear Protein Regulation, School of Basic Medical Sciences, Henan University, Kaifeng 475004, China

**Keywords:** endoplasmic reticulum stress, NLRP3 inflammasome, nonalcoholic fatty liver disease, hepatic ischemia–reperfusion, hepatotoxicity, liver injury

## Abstract

The endoplasmic reticulum (ER) is a key organelle responsible for the synthesis, modification, folding and assembly of proteins; calcium storage; and lipid synthesis. When ER homeostatic balance is disrupted by a variety of physiological and pathological factors—such as glucose deficiency, environmental toxins, Ca^2+^ level changes, etc.—ER stress can be induced. Abnormal ER stress can be involved in many diseases. NOD-like receptor family pyrin domain-containing 3 (NLRP3), an intracellular receptor, can perceive internal and external stimuli. It binds to apoptosis-associated speck-like protein containing a CARD (ASC) and caspase-1 to assemble into a protein complex called the NLRP3 inflammasome. Evidence indicates that ER stress and the NLRP3 inflammasome participate in many pathological processes; however, the exact mechanism remains to be understood. In this review, we summarized the role of ER stress and the NLRP3 inflammasome in liver disorders and analyzed the mechanisms, to provide references for future related research.

## 1. Introduction

**Figure 1:**
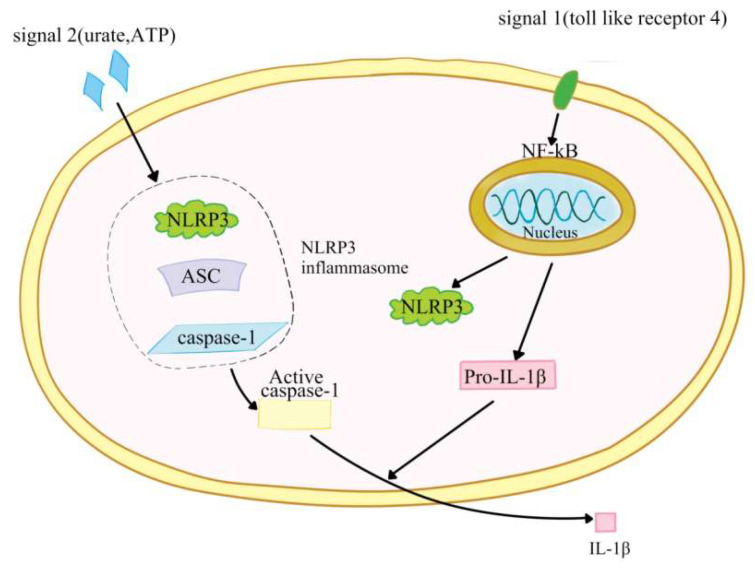
The activation of NRLP3 inflammasome.

The definition of inflammasomes was first proposed by Tschopp et al. in 2002 [1]. Inflammasomes are a group of cellular protein complexes, which can recognize exogenous microorganisms, endogenous danger signals and different stressors; as a response, they activate caspase-1 to produce IL-1β and IL-18 to initiate inflammation [2,3]. So far, inflammasomes have been found to include nucleotide-binding domain leucine-rich repeat (NLR) and pyran domain-containing receptor 1 (NLRP1); NLRP3; RIG-I; and caspase recruitment domain containing receptor 4 (NLRC4); and they have been found to be absent in melanoma 2 (AIM2) [3,4]. NLRP3 inflammasome is the most thoroughly studied one at present, and is composed of NLRP3, apoptosis-associated speck-like protein (ASC) and pro-caspase-1 precursor [5,6,7,8,9]. NLRP3, a 115 kDa cytoplasmic protein, contains three domains: one is a leucine-rich repeat (LRR) at the C-end; the second is a central nucleotide-binding and oligomeric domain NACHT with ATPase activity, and the third is a pyran domain (PYD) at the N-end, which is used to recruit ASCs [10]. NLRP3 is expressed in monocytes, dendritic cells, neutrophils, epithelial cells, osteoblasts and lymphocytes [11]. ASC contains an amino terminal PYD and a carboxyl terminal CARD. Under specific stimulation, ASC interacts with NLRP3 through the PYD–PYD domain [3]. ASC recruits pro-caspase-1 through CARD–CARD domain interactions [12,13]. Under exogenous or endogenous stimulation, NLRP3 in the cell is activated, interacts with pre-caspase-1 and ASC to form a large protein complex, and activates caspase-1. Activated caspase-1 converts pre-IL-1β and pre-IL-18 into IL-1β and IL-18, which promotes inflammation by inducing the production of pro-inflammatory cytokines, chemokines and growth factors [14]. The activation of NRLP3 inflammasome requires two steps. The first step is induced by the first signal (signal 1) including toll-like receptor 4 and many endogenous risk signals, which activates NF-ĸB to upregulate the expression of NLRP3, pro-IL-1β, and pro-IL-18 [15,16]. The second step is the activation step and is induced by a signal (signal 2) including urate, extracellular adenosine triphosphate (ATP) and cholesterol crystals. Signal 2 promotes NLRP3 inflammasome assembly and activates caspase-1 to convert pre-IL-18 and IL-1β into their active forms [17] (Figure 1). It will lead to host inflammatory injury when NLRP3 inflammasome is overactivated [18]. Therefore, abnormal NLRP3 inflammasome can be involved in a variety of diseases, including liver diseases [19,20,21,22,23].

The endoplasmic reticulum (ER) is an organelle responsible for the synthesis, folding and modification of the secretion/transmembrane protein. It also plays a key role in lipid calcium storage, detoxification and biosynthesis [17,24,25]. When many physiological and pathological factors—including glucose deficiency, environmental toxins, Ca^2+^ level changes, viral infection, oxidative stress, inflammation and hypoxia–disrupt ER homeostatic balance, ER stress can be induced, forming a large number of unfolded and misfolded proteins, calcium depletion and lipid synthesis disorders [26,27]. Cells can reduce the damage of misfolded protein and alleviate the stress state in two ways. One is that ER stress triggers the unfolded protein reaction (UPR) to reduce the synthesis of new proteins and increase the expression of molecular chaperones that can promote protein folding. The second is to increase the degradation of misfolded proteins [28]. ER stress-induced UPR is mediated by three parallel signaling pathways: the activated transcription factor 6 (ATF6)-mediated pathway; the inositol dependent enzyme 1 (IRE1)-mediated pathway; and the pancreatic endoplasmic reticulum kinase (PERK)-mediated pathway [29]. Moderate ER stress can promote the recovery of ER homeostasis to help cells adapt to environmental changes. Excessive ER stress can induce caspase-12-dependent apoptosis, thus leading to many diseases [30]. Previous studies have shown that ER stress is involved in the occurrence and development of many disorders including diabetes, obesity, cancer, inflammation, neurodegenerative diseases and autoimmune diseases [31,32,33,34,35]. It has also been reported that ER stress and the NLRP3 inflammasome participate in many physiological and pathological processes; however, the exact mechanisms are unclear [36]. In this review, we summarized the role of ER stress and NLRP3 inflammasome in liver disorders and analyzed the mechanisms, to provide references for future related research.

## 2. The Role of Endoplasmic Reticulum Stress and the NLRP3 Inflammasome in Nonalcoholic Fatty Liver Disease

Nonalcoholic fatty liver disease (NAFLD) is a clinicopathological syndrome characterized by the accumulation of liver fat, excluding viral infection and excessive drinking, and includes fatty liver, nonalcoholic steatohepatitis, and liver cirrhosis. It is a common chronic liver disease in the world. Due to its high incidence rate (about 20–30%) and long-term clinical treatment, NAFLD has become a serious public health problem [37,38,39]. Many risk factors are related to the progress of NAFLD, such as type 2 diabetes, visceral obesity, hyperlipidemia, and insulin resistance, but the exact pathogenesis is not yet fully understood [40]. At present, insulin sensitizers, antioxidants, lipid-lowering drugs and liver-protecting drugs are mainly used to treat NAFLD; however, the therapeutic effects are poor, and some drugs are found to have obvious toxic side effects, and can cause great harm to patients [41]. So far, no effective method for the prevention and treatment of NAFLD has been found. Therefore, it is particularly important to deeply study the pathogenesis of NAFLD and explore its effective treatment.

### 2.1. The Role of Endoplasmic Reticulum Stress and NLRP3 Inflammasome in Nonalcoholic Steatohepatitis

Nonalcoholic steatohepatitis (NASH) is the combination of lipid accumulation, hepatocyte death, inflammation, and fibrosis, which can develop into advanced fibrosis and hepatocellular carcinoma [42,43]. It is unclear how hepatic steatosis transforms into NASH [43]. It has been reported that the ER stress in hepatocytes may be related to the development of steatosis to NASH [44,45]. The results of C Lebeaupin et al. showed that tauroursodeoxycholic acid (TUDCA), an ER stress inhibitor, could inhibit balloon degeneration, apoptosis, and inflammasome activation of hepatocytes in obese mice that have severe steatosis and are stimulated by LPS. In the liver of obese mice, treatment with LPS or tunicamycin (an ER stress inducer) resulted in the activation of IRE1α, PERK and CHOP overexpression, which activated the NLRP3 inflammasome, subsequently triggering caspase-1, caspase-11, interleukin-1β-mediated hepatocyte pyroptosis, and caspase-3-dependent apoptosis. Meanwhile, TUDCA could abolish the above changes caused by LPS, indicating that TUDCA suppressed NLRP3 inflammasome-induced pyroptotic death by inhibiting LPS-induced ER stress to improve the NASH model, and ER stress promoted NLRP3 inflammasome-induced pyroptosis. Knocking-down *Chop* using siRNA inhibited the activity of caspase-11, caspase-1 and IL-1β, but not of active caspase-3, in tunicamycin or tunicamycin + LPS-induced primary hepatocytes; this indicates that ER stress induced NLRP3 inflammasome pyroptosis to cause injury of the LPS-induced NASH model via CHOP. In conclusion, ER stress could activate the NLRP3 inflammasome and subsequent pyroptosis and apoptosis, which promoted NASH progression. Therefore, the inhibition of ER stress-dependent NLRP3 inflammasome activation and subsequent cell death might be a potential treatment for nonalcoholic hepatitis. The ER stress effectors PERK and IRE1α could both activate CHOP, thereby activating NLRP3 inflammasome, which further proved that ER stress promoted the NLRP3 inflammasome via the CHOP pathway [46]. Reactive oxygen species (ROS) can activate the NLRP3 inflammasome [47]. In the above model, LPS and tunicamycin co-treatment only caused a slight increase in ROS; therefore, further studies are needed to exclude the effect of ER stress on NLRP3 through ROS. Moreover, whether ROS can further enhance the effects of the IRE1α-PERK–HOP axes, thus exacerbating NLRP3 inflammasome-induced pyroptosis in LPS-induced hepatocyte, needs to be studied.

### 2.2. Bax Inhibitor-1 Improves NAFLD through Endoplasmic Reticulum Stress and NLRP3 Inflammasome

Bax Inhibitor-1 (BI-1) is a negative regulator of ER stress and can improve NAFLD [48,49]. Cynthia Lebeaupin and colleagues found that BI-1 gene ablation in tunicamycin-treated BI-1^−/−^ mice made the liver vulnerable to NAFLD, which led to hepatic steatosis and metabolic collapse; this was evidenced by an increase in fatty acid uptake, inhibition of β-oxidation, and a reduction in fatty acid release. Moreover, the enhanced ER stress promoted NLRP3 inflammasome activation, hepatocyte death, fibrosis, and the dysregulation of lipid homeostasis, leading to liver injury in the livers of tunicamycin-treated BI-1^−/−^ mice. In liver biopsies obtained from NAFLD patients, the activation of the IRE1α signaling pathway was accompanied by BI-1 downregulation, suggesting that the IRE1α signaling pathway contributed to NAFLD. Moreover, the enhanced ER stress evidenced by the increasing expression of liver X-box binding protein 1 (XBP1), IRE1a, and the C/EBP homologous protein (CHOP) in HFD-fed BI-1^−/−^ mice with NASH could also activate the NLRP3 inflammasome. Similarily, in primary mouse hepatocytes lacking BI-1, the IRE1α signaling pathway was shown to mediate NLRP3 inflammasome activation and cell death. Additionly, the inhibition of IRE1α signaling with STF-083010 counteracted the BI-1 deficiency promotion of NAFLD, indicating that BI-1 could improve NAFLD by suppressing ER stress-induced IRE1α-dependent NLRP3 inflammasome activation [50]. It can be seen from the above that ER stress-induced IRE1α-dependent NLRP3 inflammasome activation is involved in lipid metabolism, so it can be speculated that it may be beneficial in treating diabetes, which is worth studing.

### 2.3. Ginsenoside Rg1 Improves NAFLD through Endoplasmic Reticulum Stress and NLRP3 Inflammasome

Ginsenoside Rg1 (Rg1) is an active ingredient of natural medicine and has a variety of physiological functions, including anti-inflammatory, anti-apoptotic, anti-fibrosis, antioxidant and neuroprotective effects. It has been reported that Rg1 is involved in NAFLD [51,52,53]. Yashu Xu et al. constructed a mouse model of NAFLD by feeding mice a high-fat diet (HFD), and committed a series of experiments. The results showed that Rg1 improved NAFLD by notably decreasing liver weight, serum aspartate aminotransferase (AST), triglyceride (TG), alanine aminotransferase (ALT), and free fatty acids (FFAs), as well as alleviating liver inflammation. The above results were confirmed using a liver tissue staining experiment. Rg1 also decreased the serum level of malondialdehyde (MDA) and upregulated the expression of superoxide dismutase (SOD) and peroxisome proliferator-activated receptor-alpha (PPARα); this promoted fatty acid beta oxidation and the metabolism of FFAs and TG, indicating that Rg1 improved NAFLD by regulating lipid peroxidation. ER stress was promoted in NAFLD, which resulted in apoptosis and inflammation and lead to hepatocyte injury. On the other hand, Rg1 inhibited ER stress by downregulating the expression of CCAAT/enhancer binding protein (C/EBP) homologous protein (CHOP), caspase 12, and glucose-regulated protein 78 (GRP78). Moreover, the NLRP3 inflammasome level and the subsequent production of interleukin 1-beta (IL-1β) and interleukin 18 (IL-18) were increased in the NAFLD model of mice, while the changes decreased with Rg1. In summary, Rg1 ameliorated NAFLD by inhibiting ER stress and inflammasome activation, which needs to be further confirmed [54]. In the above study, the relationship between ER stress and NLRP3 inflammasome in the improvement of NAFLD with Rg1 remains to be further studied.

### 2.4. Acetylantroquinonol B Improves NAFLD through Endoplasmic Reticulum Stress and the NLRP3 Inflammasome

Acetylantroquinonol B (4-AAQB) is a ubiquinone from Antrodia cinnamomea and has antioxidant, anti-inflammatory and anti-hepatoma properties [55]. The results of I-Chuan Yen, et al. showed that 4-AAQB improved methionine/choline-deficient (MCD) diet-induced NASH by attenuating steatosis, immune cell filtration and hepatic ballooning, and by reducing the plasma levels of AST and ALT. Additionly, inflammation, ER stress, and NLRP3 inflammasome were all upregulated in in vitro and in vivo models, while 4-AAQB decreased these changes. 4-AAQB also activated the nuclear factor erythroid 2-related factor 2 (Nrf2) and Sirtuin 1 signaling pathways in vitro and in vivo [56]. SIRT1 inhibited hepatic inflammation, ER stress, and lipogenesis, indicating that SIRT1 could improve NAFLD [57,58,59,60] and possessed a beneficial metabolic function [61]. An SIRT1 gene knockout exacerbated palmitic acid (PA)-induced NLRP3 inflammasome activation and subsequent inflammation in AML-12 cells, indicating that 4-AAQB suppressed ERS/NLRP3 inflammasome by activating SIRT1 [62]. Studies have shown that Nrf2-deficient mice are prone to NASH [63], showing that Nrf2 is a promising therapeutic target for NAFLD [64,65]. In addition, SIRT1 is related to the activation of the Nrf2 antioxidant pathway in vivo [66]. The activation of the Nrf2 pathway improves NASH progression by inhibiting ER stress [67]. Therefore, it can be deduced that 4-AAQB ameliorated NAFLD through the inhibition of ERS/NLRP3 inflammasome by activating SIRT1-the Nrf2 pathway, which needs to be further verified. In the above study, 4-AAQB could scavenge ROS to inhibit oxidative stress [56]; therefore, in the improvement of NAFLD using 4-AAQB, ER stress may inhibit the NLRP3 inflammasome through ROS, which needs to be further studied.

In summary, at present, there are few studies on the role of ER stress/NLRP3 inflammasome in NAFLD; moreover, some studies are very superficial and can only draw preliminary conclusions, which need further research to be verified. In particular, the mechanism of ER stress/the NLRP3 inflammasome involved in liver lipid metabolism remains to be further explored. In addition, in the above studies, ER stress/the NLRP3 inflammasome were inhibited to improve NAFLD. However, whether enhanced endoplasmic reticulum stress can improve NAFLD by inhibiting NLRP3 inflammatory bodies remains to be clarified. ER stress/the NLRP3 inflammasome may become a new strategy for the treatment of NAFLD.

## 3. The Role of Endoplasmic Reticulum Stress and the NLRP3 Inflammasome in Hepatic Ischemia–Reperfusion

Hepatic ischemia–reperfusion (HIR) is a physiological and pathological phenomenon, which is difficult to avoid in some types of surgery and is related to liver transplantation, liver injury and hepatectomy [68,69]. When the blood flow is restored, the liver is subjected to new attacks due to the initial metabolic imbalance caused by the sudden supply of nutrients (especially oxygen), resulting in the deterioration of the injury. This phenomenon is called hepatic ischemia–reperfusion injury (HIRI) [70,71]. γ-Oryzanol (ORY) is one of the rice bran oil (RBO) compounds, which is known as the main food source in the world [72]. ORY has been reported to have anti-diabetic, anti-hyperlipidemic, anti-carcinogenic, anti-inflammatory, anti-ulcerogenic, and antioxidant effects [73,74]. Yichao Du and colleagues orally administered ORY to mice for 7 days, followed by liver ischemia for 60 min and reperfusion for 6 h. The results showed that ORY mitigated HIRI in mice by decreasing the serum AST and ALT levels; it also improved hemorrhagic focus, the collapse of the hepatic lobule structure, and hepatocyte necrosis in a HIRI model of rats. The in-depth research revealed that ORY upregulated the levels of GSH and SOD, and downregulated the levels of MDA and MPO in a HIRI model of rats, indicating that ORY inhibited I/R-induced lipid peroxidation, oxidative stress and neutrophil infiltration. ORY also inhibited ER stress during HIRI by reducing the expression levels of CHOP, p-PERK and GRP78. Moreover, ORY significantly decreased the protein expressions of NLRP3, caspase-1, IL-1β, and Bax, and increased Bcl-2 protein expression to protect the liver from inflammation and apoptosis induced by I/R in the rat model. Similar results were obtained in AML12 cells (mouse normal hepatocytes) in vitro. Collectively, ORY improved HIRI by suppressing the NLRP3 inflammasome and ER stress [75]. Studies have revealed that the massive production of ROS is closely related to HIRI [76]. In the above study, ORY notably reduced ROS levels in CoCl_2_-Induced AML12 cells; therefore, it can be deduced that ER stress inhibits NLRP3 inflammasome by clearing ROS. More and more evidence has proven that ER stress and NLRP3 inflammasome-mediated injury have a great impact on ischemia–reperfusion injury [77,78], which needs further study. ER stress and NLRP3 inflammasome may become a target for the treatment of hepatic ischemia–reperfusion injury in the future.

## 4. The Role of Endoplasmic Reticulum Stress and the NLRP3 Inflammasome in Hepatotoxicity

### 4.1. Allicin Improves Hepatotoxicity through Endoplasmic Reticulum Stress and the NLRP3 Inflammasome

Acrylamide (AA) is produced by a Maillard reaction during thermal processing and a well-known potential carcinogenic compound [79]. AA has been reported to have hepatotoxic effects [80]. Allicin is one of the active components in garlic bulbs [81], and has many biological benefits, including anticancer, hypolipidemic, blood-pressure-lowering, diabetes improvement, anti-hepatic-steatosis and anti-inflammation [82,83]. Allicin can inhibit AA-induced hepatocyte injury and toxicity by inhibiting intracellular ROS release and oxidative stress (OS) [84,85]. Bo Nan et al. found that allicin downregulated CYP2E1 protein expression and ROS release to finally reduce OS-induced liver injury in Kupffer cells and SD rat livers treated with AA. Meanwhile, allicin significantly decreased the expression of the ER stress marker proteins CHOP and GRP78, and the expression of IRE1α pathway key proteins TRAF2, p-ASK, p-IRE, and XBP-1s induced by AA; this indicates that allicin inhibited AA-induced ER stress. Furthermore, allicin suppressed AA-induced the MAPK and NF-κB pathways by downregulating p65, JNK, p38, ERK, and IκBα phosphorylation in Kupffer cells and SD rat livers. Additionally, allicin also decreased cleaved caspase-1 expression and the release of IL-1β, IL-6, IL-18, and TNF-α to inhibit AA-induced-NLRP3 inflammasome activation, thus mitigating AA-induced liver inflammation. Collectively, allicin can reduce AA-induced NLRP3 inflammasome activation by inhibiting ER stress and OS, thus improving AA-induced hepatotoxicity; this needs to be further verified using inhibitors, such as ER stress inhibitors and NLRP3 inhibitors [86]. It has been reported that high glucose can produce ROS and activate the MAPK and NF-κB signaling pathways to induce inflammation in HepG2 cells [87]. Additionally, ER stress can regulate the MAPK and NF-κB signaling pathways [88,89]. Therefore, it can be deduced that ER stress inhibits the NLRP3 inflammasome via the MAPK and NF-κB signaling pathways in the improvement of AA-induced hepatotoxicity using allicin, which needs to be further confirmed.

### 4.2. Baicalin Improves Hepatotoxicity through Endoplasmic Reticulum Stress and NLRP3 Inflammasome 13

Baicalin (BA) is one of the main bioactive components of the Chinese herbal medicine Scutellaria baicalensis. It has many pharmacological activities, such as antitumor, antibacterial, and antioxidant [90,91,92]. BA has been reported to be closely related to lipid metabolism; however, the exact mechanism is unclear [93,94]. The results of Junli Zhang et al. showed that 400 μM PA induced ER stress, evidenced by the elevated expression of the ER stress marker IRE1α and hyperphosphorylation in AML-12 cells. BA (12.5 μM and 25 μM) and TUDCA significantly inhibited p-IRE1α expression to suppress PA-induced ER stress. BA and TUDCA also inhibited intracellular PA-induced ROS generation and apoptosis of AML-12 cells, indicating that BA could suppress oxidative stress and apoptosis induced by ER stress. Moreover, BA and TUDCA significantly inhibited the expression of TXNIP and NLRP3 induced by PA, which was reversed by compound C (an AMPK inhibitor), indicating that BA inhibited ER stress via the TXNIP/NLRP3 pathway through the AMPK pathway. Collectively, BA improved cytotoxicity of AML-12 cells induced by PA, through the inhibition of ER stress via the TXNIP/NLRP3 pathway, through the AMPK pathway [95]. In contrast to the inhibition of ER stress by BA in the above study, Wang et al. found that BA induced apoptosis by promoting ER stress by activating the ATF6 signaling pathway in human hepatoma cells [96]. The reason may be that the basic level of ER stress differs between different types of cells. Studies have shown that ROS/TXNIP-induced activation of the NLRP3 inflammasome plays an important role in NAFLD [97,98,99]. Similarly, in the above study, it can be seen that ER stress regulates the NLRP3 inflammasome through ROS/TXNIP in BA improvement of hepatotoxicity. Targeting the TXNIP/NLRP3 pathway may be promising in liver diseases.

## 5. Farnesoid X Receptor Improves Liver Injury through Endoplasmic Reticulum Stress and NLRP3 Inflammasome

The farnesoid X receptor (FXR) is a member of the nuclear receptor family and exists in the intestine and liver. It helps to maintain systemic metabolic homeostasis by regulating glucose, bile acid, lipid metabolism, and energy homeostasis. Furthermore, the FXR also plays an important role in many organs, including the liver, cardiovascular system, kidney, intestine, breast, pancreas and brain [100,101,102,103]. Liver FXR activation has beneficial effects on metabolic disorders, such as NAFLD, diabetes mellitus and cholestasis [104,105,106]. The results of Chang Yeob Han et al. showed that, in patients with NAFLD and mice with liver injury, the level of FXR in the liver was negatively correlated with the activation of NLRP3 inflammasome, suggesting an inhibitory role of FXR on the NLRP3 inflammasome. In hepatocytes treated with tunicamycin, FXR levels significantly decreased, the levels of NLRP3, TXNIP, and cleaved caspase-1 and IL-1β increased; this indicates that ER stress-induced NLRP3 inflammasome in rat hepatocytes was related to FXR inhibition. FXR deficiency in mice cooperated with ER stress-induced NLRP3 and the thioredoxin-interacting protein (TXNIP), which aggravated liver injury. Meanwhile the treatment of wild-type mice with GW4064 (an FXR agonist) had the opposite effect, indicating that FXR inhibited ER stress-induced NLRP3 and TXNIP. Moreover, FXR suppressed NLRP3 and TXNIP via the PERK–CHOP pathway. In summary, FXR suppresses ER stress-induced NLRP3 inflammasome via the PERK–CHOP signaling pathway in hepatocytes to improve liver injury. ER stress promoted NLRP3 inflammasome via the PERK–CHOP signaling pathway [107]. TXNIP interacts with and activates NLRP3 [108,109,110]. However, in the above study, TXNIP knockdown had no influence on NLRP3, indicating that ER stress-mediated NLRP3 induction may not be related to TXNIP [107].

## 6. Conclusions

Accumulating evidence indicates that ER stress and the NLRP3 inflammasome play an important role in liver disorders. In this review, we summarized the following: (1) ER stress could activate NLRP3 inflammasome and subsequent pyroptosis and apoptosis to lead to nonalcoholic hepatitis; (2) BI-1 could improve NAFLD by suppressing ER stress-induced IRE1a-dependent NLRP3 inflammasome activation; (3) Rg1 improved NAFLD through the inhibition of ER stress and NLRP3 inflammasome activation, which needs to be further confirmed; (4) 4-AAQB ameliorated NAFLD through the inhibition of ERS/NLRP3 inflammasome by activating the SIRT1-Nrf2 pathway, which needs to be further confirmed; (5) ORY ameliorated HIRI through the inhibition of the NLRP3 inflammasome and ER stress; (6) ER stress inhibits the NLRP3 inflammasome via the MAPK and NF-κB signaling pathways in the improvement of AA-induced hepatotoxicity using allicin, which needs to be further confirmed; (7) BA improved the PA-induced cytotoxicity of AML-12 cells through the inhibition of ER stress via the TXNIP/NLRP3 pathway, through the AMPK pathway; (8) FXR suppressed the ER stress-induced NLRP3 inflammasome via the PERK–CHOP signaling pathway in hepatocytes to improve liver injury (Table 1).

**Figure 2:**
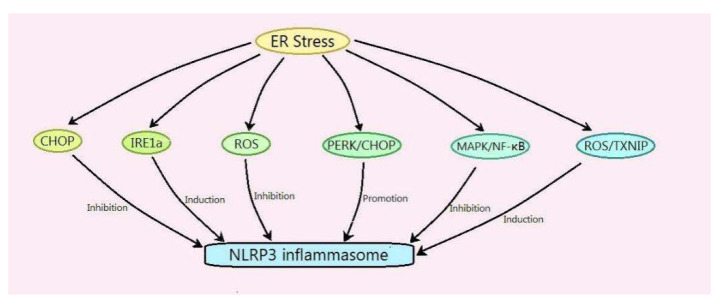
Mechanism of endoplasmic reticulum stress regulating the NLRP3 inflammasome in liver disorders.

It can be seen from the above summarized research that the mechanism of ER stress-regulating NLRP3 is as follows: (1) ER stress promotes the NLRP3 inflammasome via the CHOP pathway; (2) ER stress induces the NLRP3 inflammasome via IRE1a; (3) ER stress inhibits the NLRP3 inflammasome by clearing ROS; (4) ER stress suppresses the NLRP3 inflammasome via the MAPK and NF-κB signaling pathways; (5) ER stress induces the NLRP3 inflammasome through ROS/TXNIP; and (6) ER stress promotes the NLRP3 inflammasome via the PERK–CHOP signaling pathway (Figure 2). In the regulation of the NLRP3 inflammasome by ER stress in liver disorders, sometimes, ER stress inhibits NLRP3, and sometimes, the opposite is true. The reason may be different physiological and pathological processes, which need further study. Most of the existing studies state that ER stress regulates the NLRP3 inflammasome in the liver. Conversely, whether the NLRP3 inflammasome can regulate ER stress in the liver, and the mechanism, need to be further studied. Evidence indicates that ER stress and the NLRP3 inflammasome are both the regulative target of hydrogen sulfide (H_2_S) [111,112]. Whether H_2_S can regulate ER stress and the NLRP3 inflammasome in liver disorders is worth studying. ER stress and the NLRP3 inflammasome will become an important target for the treatment of liver disorders.

## Figures and Tables

**Table 1:** Summary of the roles of endoplasmic reticulum stress and NLRP3 inflammasome in liver disorders.

The Type of Pathological Processes	The Role of ER Stress and the NLRP3 Inflammasome	Experimental Model	Reference
Nonalcoholic hepatitis	ER stress promoted the NLRP3 inflammasome and subsequent pyroptosis and apoptosis to promote nonalcoholic hepatitis	Mouse/mouse primary hepatocyte model of nonalcoholic hepatitis	[46]
Nonalcoholic fatty liver disease (NAFLD)	BI-1 improved NAFLD through inhibition of ER stress-induced and IRE1a-dependent NLRP3 inflammasome activation	Mouse/mouse hepatocyte model of NAFLD	[50]
NAFLD	Rg1 improved NAFLD through the inhibition of ER stress and NLRP3 inflammasome activation	Mouse model of NAFLD	[54]
NAFLD	4-AAQB ameliorated NAFLD through the inhibition of ERS/NLRP3 inflammasome by activating the SIRT1-Nrf2 pathway	Male C57BL/6J mouse model of NAFLD	[56]
Hepatic ischemia–reperfusion(HIRI)	ORY ameliorated HIRI through the inhibition of the NLRP3 inflammasome and ER stress	C57BL/6 mouse model of HIRI	[75]
Hepatotoxicity	Allicin improved AA-induced hepatotoxicity through ER stress inhibition of NLRP3 inflammasome via the MAPK and NF-κB signaling pathways	Sprague Dawley rats/Kupffer cell model of hepatotoxicity	[86]
Hepatotoxicity	BA improved PA-induced cytotoxicity of AML-12 cells through the inhibition of ER stress via the TXNIP/NLRP3 pathway, through the AMPK pathway	AML-12 cell model of hepatotoxicity	[95]
Liver injury	FXR improved liver injury through inhibition of ER stress-induced NLRP3 inflammasome via the PERK–CHOP signaling pathway	C57BL/6J mouse/AML-12 cell model of liver injury	[107]

## Data Availability

Not applicable.
